# Improvement of gut-vascular barrier by terlipressin reduces bacterial translocation and remote organ injuries in gut-derived sepsis

**DOI:** 10.3389/fphar.2022.1019109

**Published:** 2022-10-07

**Authors:** Zenan Chang, Yinan Zhang, Ming Lin, Shihong Wen, Hanjin Lai, Yaqing Zhan, Xiufen Zhu, Zhikun Huang, Xuyu Zhang, Zimeng Liu

**Affiliations:** ^1^ Guangdong Clinical Research Center for Critical Care Medicine, Department of Critical Care Medicine, The First Affiliated Hospital, Sun Yat-sen University, Guangzhou, China; ^2^ NHC Key Laboratory of Assisted Circulation, Sun Yat-sen University, Guangzhou, China; ^3^ Department of Anaesthesiology, The First Affiliated Hospital, Sun Yat-sen University, Guangzhou, China; ^4^ Department of Anaesthesiology, Guangdong Provincial Hospital of Chinese Medicine, Guangzhou, Guangdong, China

**Keywords:** sepsis, gut-vascular barrier, bacterial translocation, terlipressin, PI3K

## Abstract

Gut-vascular barrier (GVB) serves as the last barrier to limit the migration of intestinal toxins into the blood circulation. The efficacy of terlipressin (a vasopressin V1 receptor agonist) in reducing GVB and multiple organ damage in gut-derived sepsis is unknown. In this study, we hypothesized that, besides other intestinal barriers, GVB play a key role in gut-derived sepsis and terlipressin improve GVB damage and then reduce bacterial translocation and organ injuries. *In vivo*, a cecal ligation and puncture mouse model was established. The mice were subjected to examine the damage of GVB determined by intestinal plasmalemma vesicle-associated protein-1(PV-1) and vascular endothelial-cadherin. And the intestinal permeability was assessed by translocation of intestinal bacteria and macromolecules. *In vitro*, transendothelial electrical resistance (TER) during interleukin (IL)-1β stimulation was measured on endothelial cells with or without small interfering RNA targeting β-catenin (si β-catenin). Terlipressin significantly improved GVB damage and reduced translocation of intestinal macromolecules and bacteria by activating PI3K signaling. Of note, intestinal PV-1 expression was significantly correlated with translocation of macromolecules, and dramatic increase of macromolecules was observed in intestinal tissues whereas fewer macromolecules and bacteria were observed in blood, liver and lung following terlipressin treatment. *In vitro*, terlipressin restored TER during IL-1β stimulation and si β-catenin transfection blocked the changes delivered by terlipressin. Collectively, terlipressin alleviated GVB damage and subsequent bacterial translocation *via* blood vessels after sepsis challenge, resulting in reduced distant organ injuries and the responsible mechanisms may involve the activation of PI3K/β-catenin pathway.

## Introduction

Sepsis is a grave multi-organ dysfunction syndrome induced by the host’s maladjusted response to infection, with a mortality rate of up to 40% (Gotts and Matthay 2016; Shankar-Hari, Phillips et al., 2016). Gut-derived infection is regarded as a leading cause of sepsis in critical ill patients (Dickson 2016; Wang, Li et al., 2019). Intestinal barrier dysfunction caused by gut-derived sepsis induces the development of the bacterial translocation and multiple organ dysfunction syndrome (MODS) (Assimakopoulos, Triantos et al., 2018). The gut-vascular barrier (GVB) is the inner layer of defense in the multiple intestinal barriers (e.g., epithelium, mucus barrier, gut microbiota) that crucially regulates the translocation of substances from the intestinal lumen to the systemic circulation (Spadoni, Zagato et al., 2015; Spadoni, Pietrelli et al., 2016; Liu et al., 2020a; Chopyk and Grakoui 2020; Paone and Cani 2020; Brescia and Rescigno 2021). Several authors indicated that, in septic rats, the intestinal vascular permeability increased (He, Yuan et al., 2018; Li et al., 2020a). To date, however, among various components of intestinal barriers, the key role of GVB in limiting bacterial translocation and distant organ injuries caused by gut-derived sepsis remains unclear.

Terlipressin, a highly selective vasopressin V1 receptor agonist, has become one of the commonly used vasoconstrictor drugs in the operating room and intensive care unit (ICU), and it successfully used in cases of septic shock (O’Brien, Clapp et al., 2002), hepatorenal syndrome (Uriz, Ginès et al., 2000), and gastrointestinal bleeding (Favalli, De Franceschi et al., 2004). Moreover, several clinical experiments are now investigating the impact of terlipressin on variceal hemorrhage (Poudel, Dhibar et al., 2022), post-hepatectomy (Li et al., 2020b), cirrhosis and ascites (Israelsen, Dahl et al., 2020). In our multicenter clinical trial, terlipressin effectively maintained the stability of circulation in the patients with septic shock (Liu, Chen et al., 2018). Importantly, we previously demonstrated that, *in vivo* and *in vitro*, terlipressin could protect against organ injury through phosphatidylinositol 3-kinase (PI3K) pathway following intestinal ischemia/reperfusion (I/R) attack (Liu, Zhang et al., 2017; Liu et al., 2020a). Although the previous studies have demonstrated that terlipressin improved intestinal microcirculation and organ functions in animals with endotoxemia (Lange, Ertmer et al., 2011; Qiu, Huang et al., 2014), the efficacy of terlipressin in reducing GVB and multiple organ damage in gut-derived sepsis is unknown.

Therefore, we hypothesized that, besides other intestinal barriers, GVB play a key role in gut-derived sepsis and terlipressin improve GVB damage and then reduce bacterial translocation and organ injuries. To test this hypothesis, we examined the change of GVB, the translocation of intestinal bacteria and macromolecules, and the impairments of multiple organs after sepsis attack *in vivo* and vitro, and explored the potential signaling related to the protective effect of terlipressin.

## Materials and methods

### Animals and operative procedures

The current animal protocol has been reviewed and approved by the Institutional Animal Care and Use Committee (IACUC) of Sun Yat-sen University (Guangzhou, China; Approval No. SYSU-IACUC-2021-000570). C57BL/6J male mice (8–12 weeks) were provided by Experimental Animal Center of Sun Yat-sen University. The mice were allowed free access to water and food.

Sepsis model was established by cecal ligation and puncture (CLP) as previous described (Rittirsch, Huber-Lang et al., 2009). Briefly, the mice were anaesthetized with pentobarbital sodium (50 mg kg^−1^, intraperitoneally). The abdominal cavity was opened by a midline laparotomy. The cecum was exposed and ligated at 50% of the whole length. A needle (20 G) was used to penetrate the cecum once at the vascular-less part of the ligated segment and a drop of intestinal content was extruded. Then, the cecum was carefully replaced and the abdominal wall was closed. Immediately after the operation, 1 ml normal saline (NS) preheated at 36°C was injected subcutaneously (Rittirsch, Huber-Lang et al., 2009). In the sham procedure, the cecum was exposed without penetration.

### Cell culture

Human umbilical vein endothelial cells (HUVECs; Sciencell) were cultured with endothelial culture medium (ECM; 1001, Sciencell) containing 5% fetal bovine serum, 1% streptomycin/penicillin solution in T75. Adherent endothelial cells were cultured in six-well plates and incubated with 10 ng/ml recombinant human interleukin-1β (IL-1β) for 24 h to mimic sepsis *in vitro* (Zhong, Wu et al., 2020).

### Animal groups and treatment

Experiment 1 To determine the change of GVB after CLP, mice were randomly allocated into five groups. In the CLP (CLP-6 h, CLP-24 h, CLP-48 h and CLP-72 h) groups, the mice were sacrificed 6 h, 24, 48, and 72 h after CLP operation respectively (*n* = 6 each). In the Baseline group, mice underwent the sham procedure and were sacrificed immediately after the procedure. Then, an indicated time point was selected for Experiment 2 according to the worst outcome of GVB damage after CLP (Supplementary Figure S1A).

Experiment 2 To investigate the effects of terlipressin on GVB and organ injuries after CLP, the mice were randomly divided into 4 groups (Control, CLP, TP, TP + LY, *n* = 8 each for “leakage test of macromolecules” test and *n* = 6 each for other tests). Control group: The mice underwent the sham procedure, and were injected intraperitoneally with 1 ml NS, and 5% dimethylsulfoxide (DMSO) and 95% Corn oil (8001-30-7, MedChemExpress, New Jersey, United States) in a total volume of 0.4 ml at 5 min after procedure. CLP group: The mice underwent the CLP procedure, and were injected intraperitoneally with 1 ml NS and 0.4 ml 5% DMSO and 95% Corn oil mixture at 5 min after CLP. TP group: The mice underwent the CLP procedure, and were injected intraperitoneally with 0.15 mg kg^−1^ terlipressin (Hybio Pharmaceutical Co., Shenzhen, China) dissolved in 1 ml NS, and 0.4 ml 5% DMSO and 95% Corn oil mixture at 5 min after CLP. TP + LY group: The mice underwent the CLP procedure, and were injected intraperitoneally with 0.15 mg kg^−1^ terlipressin dissolved in 1 ml NS at 5 min after CLP. Meanwhile, 40 mg kg^−1^ LY294002 (the specific inhibitor of PI3K, S1105, Selleck Chemicals, Houston, Texas, United States) dissolved in 0.4 ml 5% DMSO and 95% Corn oil mixture was also injected (Liu, Li et al., 2019). The mice were killed at the indicated time point and the biological samples were collected (Supplementary Figure S1B).

*In vitro*, to determine the effect of terlipressin on the cultured HUVECs, the cells were divided into six groups. Control group: HUVECs were treated without any treatment. IL-1β group: HUVECs were subjected to 10 ng/ml recombinant human IL-1β for 24 h. IL-1β+TP (25 nM) and IL-1β+TP (100 nM) groups: HUVECs were treated with 10 ng/ml recombinant human IL-1β and terlipressin at concentration (25 nM or 100 nM) for 24 h. IL-1β+TP + si β-catenin and IL-1β+TP + si Negative control (NC) groups: Small interfering RNA (si β-catenin or NC) was transfected before HUVECs treating with IL-1β and terlipressin.

### Leakage test of macromolecules

To investigate the permeability of GVB, 0.5 g kg^−1^ 70kd-Fluorescin Isothiocyanate (FITC)-dextran (FD70, 60842-46-8, Sigma-Aldrich, St. Louis, United States), which cannot pass through the normal vascular wall (Spadoni, Zagato et al., 2015), was diluted in 0.5 ml PBS before anesthesia and then administered intragastrically to the independent mice because the usage of FD70 disturbed the detections of other variables. The optical density (OD) of FD70 in serum was read by a multi-label analyzer (INFINITE F500, Tecan, Austria) (Obermüller, Frisina et al., 2020). The ileum, colon, liver and lung tissue were sectioned and the nuclei were counterstained with 4′,6-diamidino-2-phenylindole (DAPI) and the images were obtained with an automatic inverted fluorescence microscope (Leica DMI8, Germany).

### Morphometric assessment

Hematoxylin-eosin (HE) staining of ileum, liver and lung was performed to evaluate the histopathological injury. Images were obtained with Olympus BX63 (Japan) microscope. Chiu’s score and Eckhoff’s score were used to evaluate the histopathological injury of intestine and liver respectively (Chiu, McArdle et al., 1970; Wen, Li et al., 2020). Lung injury score was carried out based on the previous literature (Li et al., 2020c). All the scoreing were evaluated by two experienced pathologists who were blinded to group allocation.

### Serum biochemical markers and cytokines detection

Serum alanine aminotransferase (ALT) and aspartate aminotransferase (AST) were detected by automatic biochemical analyser (Chemray 800, Shenzhen, China). Serum interleukin (IL)-6 and lipopolysaccharides (LPS) were detected by enzyme-linked immunosorbent assay (ELISA) kits (CSB-E04639m and CSB-E13066m, CUSABIO, Wuhan, China).

### Immunofluorescence

Immunofluorescence was used to detect the co-localization of endothelial marker (CD31) and other proteins. Primary antibodies included CD31 (1:400, ab24590, Abcam, Cambridge, United Kingdom), PV-1 (1:50, ab27853), β-catenin (1:400, ab16051) and vascular endothelial (VE)-cadherin (1:250, 555289, BD Pharmingen, New Jersey, United States), incubating overnight in a wet chamber at 4°C. Secondary antibodies included goat anti-mouse (1:200, Alexa Fluor 647, ab150115), goat anti-rat (1:200, Alexa Fluor 488, ab150157), and goat anti-rabbit (1:400, BS-0295G-FITC, Bioss, Beijing, China), incubating at room temperature in dark for 1 h. Then the nuclei were counterstained with DAPI and images were obtained with an automatic inverted fluorescence microscope (Leica DMI8, Germany).

### Fluorescence *in situ* hybridization

Specimens were incubated with hybridization buffer at 37°C for 1 h. The pre-hybridization solution was removed, and the EUB338: 5′- GCT GCC TCC CGT AGG AGT -3′ bacterial probe labeled by Cy3 (red) was added to detect bacterial translocation. Sections were incubated with DAPI for 8 min in the dark, and then mounting with anti-fluorescence quenching sealing tablets.

### Quantitative real-time polymerase chain reaction

The mRNA levels of *IL-6, IL-1β, TNF-α*, *PV-1, β-catenin, VE-cadherin, occludin and zonula occludens 1 (Z O -1)* were determined by q-PCR. After homogenate or scraping cells, HP Total RNA Kit (R6812-02, Omega, United States) was used to extract total RNA from tissue samples. RNA was reversely transcribed into cDNA with HiScript II Q RT Supermix for qPCR (R222-01, Vazyme, Nanjing, China). Q-PCR was performed with SYBR qPCR Master Mix (Q711-02, Vazyme, Nanjing, China) on Light Cycler 480 (Roche, Switzerland). The levels of target genes relative to *β-actin* were calculated by ∆∆CT method. Primer sequences were listed in Supplementary Table S1.

### Western blot analysis

After homogenate or scraping and centrifugation at 12,000 rpm⋅min^−1^ for 20 min, the concentration of supernatant was determined by bicinchoninic acid method (BCA, C0020, SolarBio, Beijing, China). 50 ug total protein was electrophoresed on poly acrylamide gels and transferred to PVDF membrane. The membranes were blocked with 5% BSA at room temperature for 1 h. The primary antibodies included PV-1 (1:1000), VE-cadherin (1:500), β-actin (1:5000, 66009-1-Ig, Proteintech, Chicago, United States), phospho-Akt (Ser473, 1:1000, 9271, CST, Danvers, United States), Akt (1:1000, CST: 9272), phospho-β-catenin (Ser33/37/Thr41, 1:1000, CST: 9561), β-catenin (1:1000), phospho-GSK-3β (Ser9, 1:1000, CST: 9336), GSK-3β (1:1000, CST: 9315) and GAPDH (1:5000, FD0063, Fude Bio, Hangzhou, China). The membranes were incubated overnight with the primary antibodies in a shaking table at 4°C and then incubated with secondary antibodies for 1 h at room temperature, including HRP-labeled goat anti-mouse IgG (1:5000, GB23301, ServeBio, Wuhan, China), HRP-labeled goat anti-rat IgG (1:5000, GB23302, ServeBio) or HRP-labeled goat anti-rabbit IgG (1:5000, FD0128, Fude Bio). After washing with tris buffered saline tween, the images were obtained on chemiluminescence instrument (Amersham Imager 600, United States) and analyzed with ImageJ software. The bands were normalized with the housekeeping proteins β-actin or GAPDH and then presented as the relative value to Baseline or Control group.

### Survival analysis

Independent mice underwent the same procedure of Experiment 2 (Supplementary Figure S1B) and were used to evaluate survival time. After Sham or CLP operation, the mice were immediately transferred to their individual cages and allowed free access to water and food for 72 h.

### Small interfering RNA transfection

The sense strand sequences of siRNA targeting β-catenin were: 5′-CAG​TTG​TGG​TTA​AGC​TCT​T-3′ (si β-catenin). The siRNA duplexes and scrambled siRNA (si Negative Control, si NC) were synthesized and purified by Tsingke (Beijng, China). siRNA transfection was performed using Lipofectamine 2000 (Invitrogen) for 24 h according to the manufacturer’s instructions.

### Transendothelial electrical resistance

The integrity of endothelial cell monolayer was quantified by transendothelial electrical resistance (TER) assay in hanging six-well plates (SPLInsert™ Hanging, 6 Inserts, Korea) by Volt/Ohm Meter for Epithelium (RE1600, jingong hongtai, Beijing, China). 5 × 104 HUVECs were grown on a transwell insert until confluency. After sterilizing, drying, and rinsing, the long ends of the electrode bridges was carefully placed into the basal chamber and the short ends was placed into the apical chamber. The longer electrode was touched the bottom of the dish, while keeping the shorter electrodes below the surface of the media but above the tissue culture inserts. The computational formula was Unit Area Resistance (Ω·cm^2^) = Resistance (Ω) × effective membrane area (cm^2^).

### Transwell permeability assay

5 × 10^4^ HUVECs were grown on a transwell insert (0.4 um pore size; SPLInsert™ Hanging, 6 Inserts) until confluency. At 24 h after treatment in each group, the supernatant was removed and FITC-dextran (1 mg/ml; 70 kDa; 60842-46-8, Sigma-Aldrich, St. Louis, United States) was added to the transwells. After 2 h, the FITC-dextran translocated to the lower compartment of the transwell was measured in a microplate reader (Thermo Scientific ™ Varioskan ™ LUX, United States) at excitation/emission wavelength of 490/520 nm. As a positive control a transwell without cells was used. By normalizing the fluorescence signals of the treatment group to the Control group a measure of endothelial layer leakiness was obtained.

### Statistical analysis

The sample size analysis was performed based on our literature (Zhang, Chang et al., 2022) and the Power and Sample Size online software (http://powerandsamplesize.com/Calculators/). A minimum of six mice per group was required for 90% power to detect a mean difference between groups of 40% in the relative expression of PV-1, assuming type I error = 0.05 for a 2-sided hypothesis test. Survival time from the beginning of CLP was expressed as median (range) and compared by Kaplan-Meier curve with Log-rank test. The mortality was analyzed by Fisher exact test. The other data were analyzed by GraphPad Prism 9.3 software (La Jolla, CA, United States) and were distributed normally. Then the values were expressed as mean ± standard deviation (SD). One-way ANOVA (Tukey post hoc) was used for comparisons among groups. Pearson correlation analysis was performed to calculate correlation coefficient and *p* value. *p* < 0.05 was considered statistically significant.

## Results

### Cecal ligation and puncture induced inflammation and organ injuries

As shown in Supplementary Figures S2A,B, LPS and IL-6 concentration in serum increased after CLP. The mRNA expression of IL-6, IL-1β and TNF-α were higher in the ileum, liver and lung after CLP insult (Supplementary Figure S2C). Furthermore, obvious intestinal damages were detected in CLP groups as evidenced by higher Chiu’s scores and depressed villus height of ileum (Supplementary Figure S2D). The injury score of liver and lung were also higher in CLP groups than those in Baseline group (Supplementary Figure S2E). These results showed that CLP procedure successfully induced gut-derived sepsis in the present study.

### Cecal ligation and puncture resulted in gut-vascular barrier damage

**FIGURE 1:**
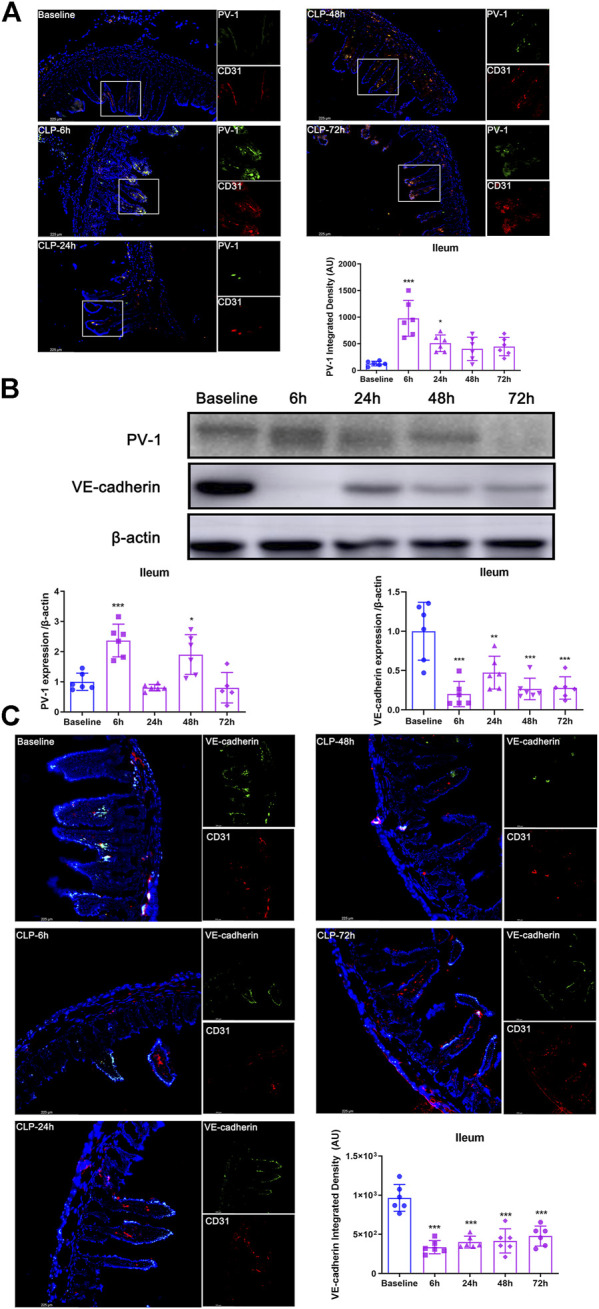
CLP resulted in GVB damage. **(A)** Immunofluorescence bimolecular CD31 (red, a marker of endothelial cell) and PV-1 (green) in the ileum (×100). Nuclei were counterstained with DAPI (blue). Scale bars: 225 μm. Quantitative analysis: integrated fluorescence density of PV-1, *n* = 6. **(B)** Expression of PV-1 and VE-cadherin in ileum after CLP operation by using western blot analysis, *n* = 6. **(C)** Immunofluorescence: CD31 (red), VE-cadherin (green) and DAPI (blue), ×200. Quantitative analysis: integrated fluorescence density of VE-cadherin, *n* = 6. Data were expressed by mean ± SD. *p < 0.05, **p < 0.01, ***p < 0.001 vs. Baseline group.

The data of immunofluorescence and western blot analysis showed that, in mice’s ileum, PV-1 expression (the specific biomarker of GVB damage) were significantly increased at 6 h after CLP (both *p* < 0.001; Figures 1A,B) and then gradually decreased. Moreover, VE-cadherin expression (another specific biomarker of GVB) significantly decreased after CLP (all *p* < 0.01; Figures 1B,C). Based on the changes of PV-1 and VE-cadherin, the critical time point (6 h after CLP) was selected for the subsequent experiments.

### Terlipressin alleviated the gut-vascular barrier damage after cecal ligation and puncture

**FIGURE 2:**
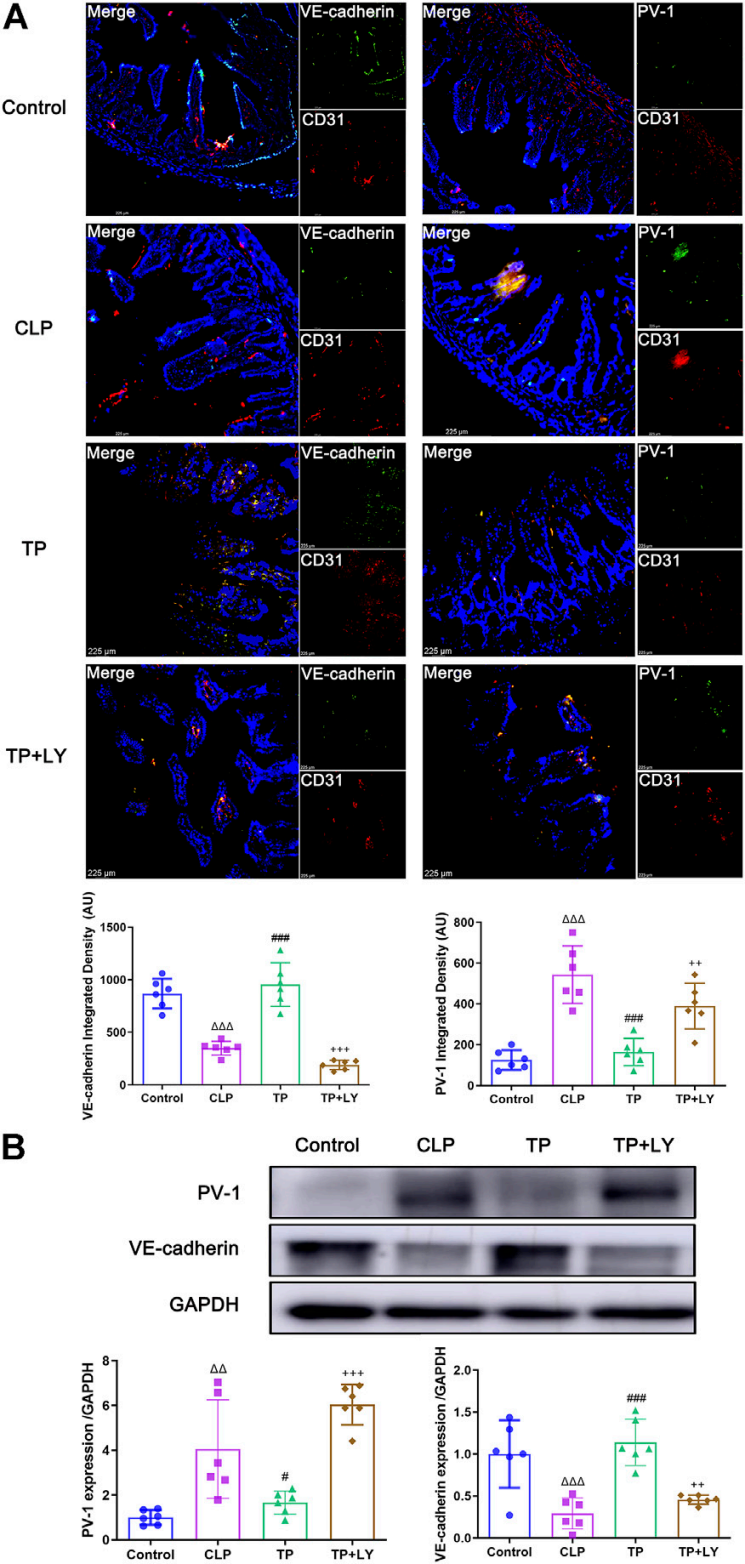
Terlipressin alleviated the GVB damage after CLP. **(A)** Dual immunofluorescence of CD31 (red) and VE-cadherin (green) or PV-1 (green) in the ileum (×200) and quantitative analysis. Nuclei were counterstained with DAPI (blue). Scale bars: 225 μm. Quantitative analysis: integrated fluorescence density of VE-cadherin and PV-1, *n* = 6. **(B)** Western blot of PV-1 and VE-cadherin in ileum and quantitative analysis, *n* = 6. Data were expressed by mean ± SD. ^Δ^ *p* < 0.05, ^ΔΔ^ *p* < 0.01, ^ΔΔΔ^ *p* < 0.001 vs. Control group. ^#^ *p* < 0.05, ^##^ *p* < 0.01, ^###^ *p* < 0.001 vs. CLP group. ^+^ *p* < 0.05, ^++^ *p* < 0.01, ^+++^ *p* < 0.001 vs. TP group.

At 6 h after CLP, the colocalization analysis of VE-cadherin or PV-1 with CD31 revealed that terlipressin restored VE-cadherin and PV-1 expression in the intestinal vascular endothelium in the TP group (both *p* < 0.001 vs. CLP group; Figure 2A). Similarly, western blot analysis indicated that the expressions of VE-cadherin and PV-1 protein were significantly improved in the TP group (TP vs. CLP group: 1.663 ± 0.5141 vs. 4.054 ± 2.202, *p* = 0.0147 for PV-1 and 1.139 ± 0.2755 vs. 0.2932 ± 0.1854, *p* = 0.0001 for VE-cadherin; Figure 2B).

### Terlipressin attenuated the translocation of intestinal macromolecules and bacteria after cecal ligation and puncture

**FIGURE 3:**
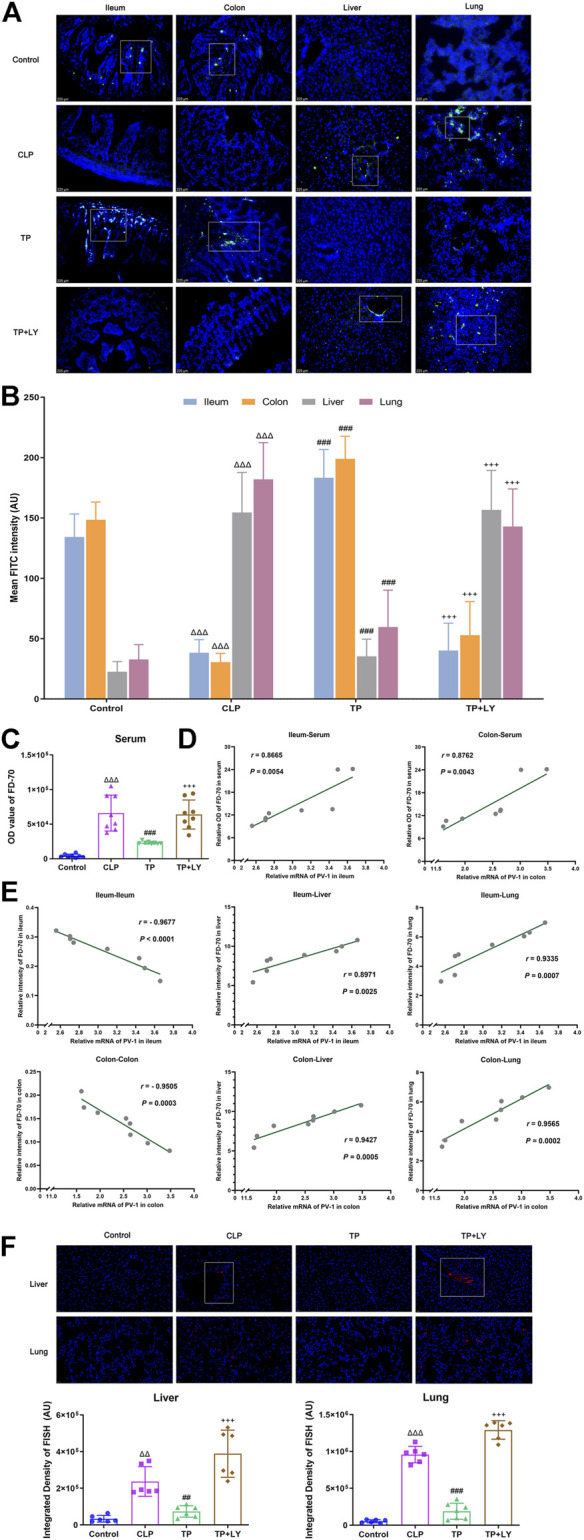
Terlipressin attenuated the translocation of intestinal macromolecules and bacteria after CLP. **(A)** Distributions of FD70 (green) in ileum, colon, liver and lung (×200). Nuclei were counterstained with DAPI (blue). Scale bars: 225 μm. **(B)** Quantitative analysis: fluorescence density of FITC, *n* = 8. **(C)** The OD value of FD-70 in serum, *n* = 8. **(D)** The correlation analyses between the mRNA expression of PV-1 in intestines and the value of FD70 in serum, *n* = 8. **(E)** The correlation analyses between the mRNA expression of PV-1 in intestines and the FD70 intensity in various organs, *n* = 8. The above analyses of migrating FD70 were performed in the independent mice. **(F)** Broad-spectrum bacteria were labeled by using a specific probe for broad-spectrum bacteria rRNA EUB338 (red), and nuclei were counterstained with DAPI (blue) in liver and lung (×400). Scale bars: 200 μm. Quantification of integrated fluorescence density of Cy3 in liver and lung, *n* = 6. Data were expressed by mean ± SD. ^Δ^ *p* < 0.05, ^ΔΔ^ *p* < 0.01, ^ΔΔΔ^ *p* < 0.001 vs. Control group. ^*#*^ *p* < 0.05, ^##^ *p* < 0.01, ^###^ *p* < 0.001 vs. CLP group. ^+^ *p* < 0.05, ^++^ *p* < 0.01, ^+++^ *p* < 0.001 vs. TP group.

The analysis of migrating macromolecules was performed in the independent mice. At 6 h after CLP, the fluorescence intensity of FD70 in intestines in the Control group were higher than those in the CLP group, whereas the value of FD70 in liver, lung and serum were lower (all *p* < 0.001, Figures 3A–C). Interestingly, terlipressin dramatically increased the FD70 intensity in ileum and colon but decreased FD70 value in liver, lung and serum (all *p* < 0.001 vs. CLP group). In the CLP group, the data of correlation analysis indicated that the mRNA level of PV-1 in the ileum and colon were positively correlated with the OD value of FD70 in serum respectively (Figure 3D). Moreover, in the CLP group, the mRNA level of PV-1 in the ileum and colon were positively correlated with the FD70 intensity in the liver and lung whereas were negatively correlated with the FD70 in the ileum or colon, respectively (Figure 3E).

The data of FISH detection showed that, in the CLP group, increased bacterial colonization was presented around blood vessels in liver and lung. Terlipressin significantly alleviated bacterial colonization in liver and lung (both *p* < 0.01 vs. CLP group, Figure 3F).

### Terlipressin reduced organ injuries and improved survival after cecal ligation and puncture

**FIGURE 4:**
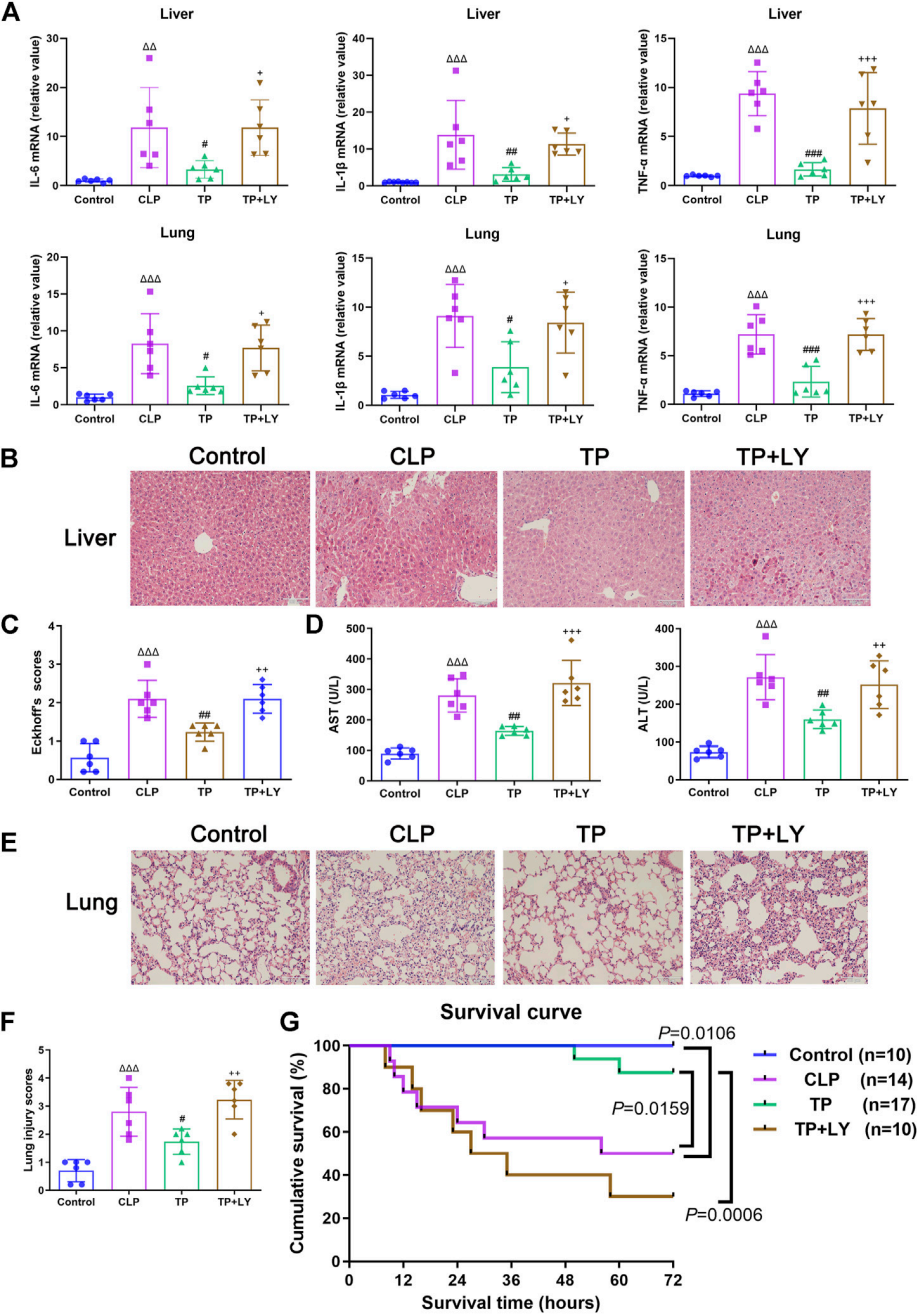
Terlipressin reduced organ injuries and improved survival after CLP. **(A)** IL-6, IL-1β and TNF-α mRNA levels in liver and lung, *n* = 6. **(B,C)** The representative images (×200) and morphological injury scores of liver, *n* = 6. Scale bars: 100 μm. **(D)** AST and ALT levels in serum, *n* = 6. **(E,F)** The representative images (×200) and morphological injury scores of lung, *n* = 6. Scale bars: 100 μm. Data were expressed by mean ± SD. **(G)** Survival analysis of different groups. ^Δ^ *p* < 0.05, ^ΔΔ^ *p* < 0.01, ^ΔΔΔ^ *p* < 0.001 vs. Control group. ^#^ *p* < 0.05, ^##^ *p* < 0.01, ^###^ *p* < 0.001 vs. CLP group. ^+^ *p* < 0.05, ^++^ *p* < 0.01, ^+++^ *p* < 0.001 vs. TP group.

At 6 h after CLP, terlipressin decreased the mRNA expressions of inflammatory cytokines in liver and lung (all *p* < 0.05 vs. CLP group, Figure 4A). Meanwhile, terlipressin treatment reduced histological injury in liver and lung and improved liver function (all *p* < 0.01 vs. CLP group, Figures 4B–F).

As shown in Figure 4G, the survival time of mice and mortality rate at 72 h after CLP in the CLP group was 64 h (10–72 h) and 50%, respectively (both *p <* 0.05 vs. Control group). Terlipressin significantly prolonged survival time which was 72 h (50–72 h; *p* = 0.0159 vs. CLP group) and decreased mortality (11.76%, *p* = 0.0439).

### Terlipressin protected against cecal ligation and puncture challenge through phosphatidylinositol 3-kinase signaling

**FIGURE 5:**
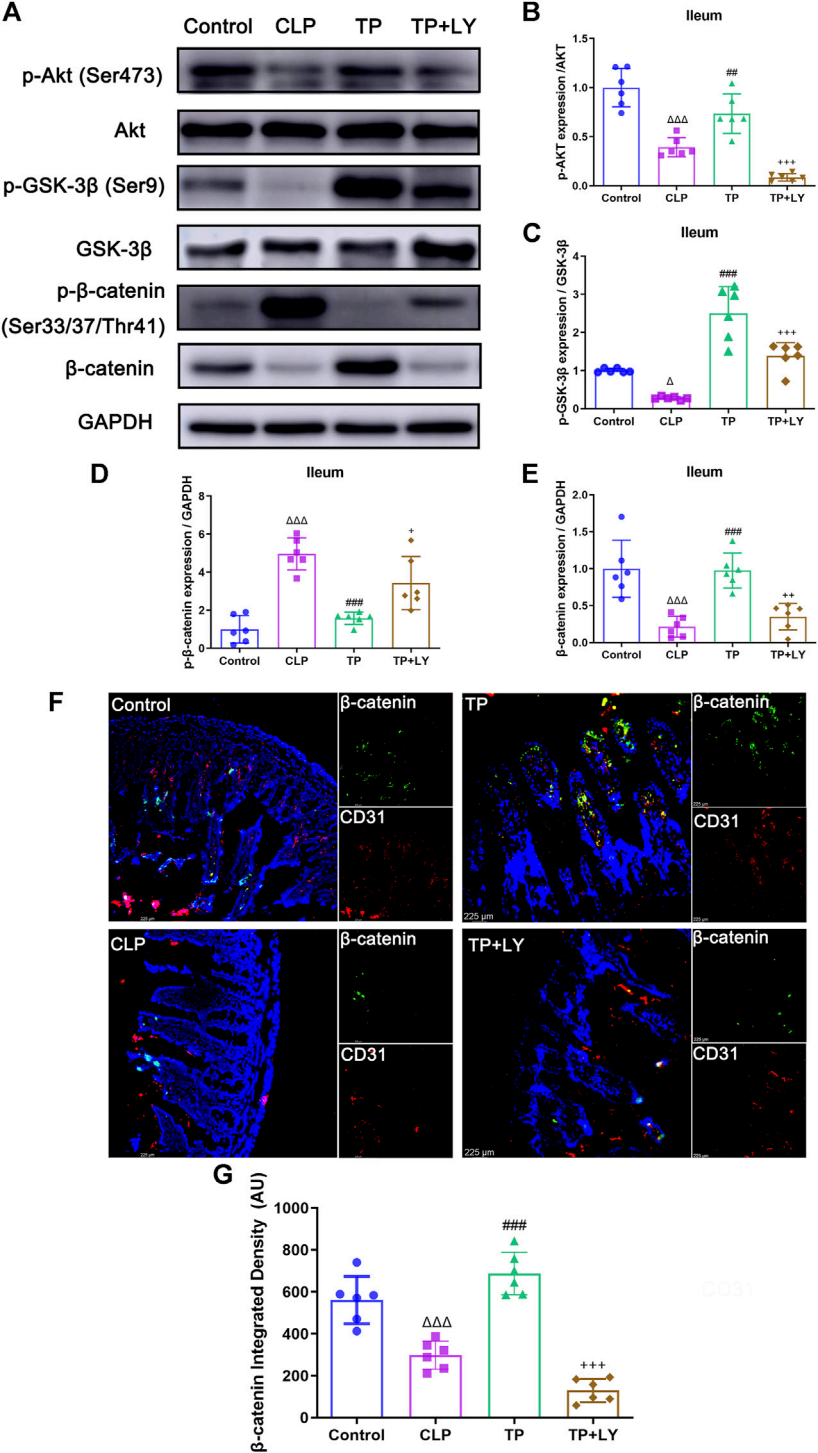
Terlipressin protected against CLP challenge through PI3K signaling. **(A)** Representative bands of p-Akt, Akt, p-GSK-3β, GSK-3β, p-β-catenin and β-catenin. **(B–E)** Quantitative analysis of above-mentioned molecules, *n* = 6. **(F)** Dual immunofluorescence of CD31 (red) and β-catenin (green) in the ileum (×200), and nuclei were counterstained with DAPI (blue). Scale bars: 225 μm. **(G)** Quantitative analysis: integrated fluorescence density of β-catenin, *n* = 6. Data were expressed by mean ± SD. ^Δ^ *p* < 0.05, ^ΔΔ^ *p* < 0.01, ^ΔΔΔ^ *p* < 0.001 vs. Control group. ^#^ *p* < 0.05, ^##^ *p* < 0.01, ^###^ *p* < 0.001 vs. CLP group. ^+^ *p* < 0.05, ^++^ *p* < 0.01, ^+++^ *p* < 0.001 vs. TP group.

To investigate the role of PI3K signaling in the GVB protection conferred by terlipressin after CLP, several molecules related to PI3K pathway were examined and LY294002, a classic inhibitor of PI3K, was used following terlipressin treatment. The results showed that, at 6 h after CLP, phosphorylation of Akt (Ser473, p-Akt) decreased after CLP (*p* < 0.0001 vs. Control group), and terlipressin significantly restored the expression p-Akt (*p* = 0.0039 vs. CLP group, Figures 5A,B). GSK-3β and β-catenin are the downstream molecule of Akt. The expression of p-GSK-3β (Ser 9) and β-catenin in ileum significantly decreased whereas p-β-catenin (Ser33/37/Thr41) increased in the CLP group (all *p* < 0.05 vs. Control group, Figures 5A,C–G). The use of terlipressin obviously increased the expression of p-GSK-3β and β-catenin in ileum (all *p* < 0.05 vs. Control group).

As shown in Figure 5, LY294002 abolished the impacts of terlipressin on the above-mentioned molecules (all *p* < 0.05 vs. TP group). Moreover, LY294002 totally diminished the protective effects of terlipressin on the GVB damage (Figure 2), the translocation of macromolecules and bacteria (Figure 3), organ injuries and survival (Figure 4) in mice after CLP (all *p* < 0.05, TP + LY group vs. TP group).

### Terlipressin maintained the integrity of endothelial cell monolayer during IL-1β stimulation *via* β-catenin

**FIGURE 6:**
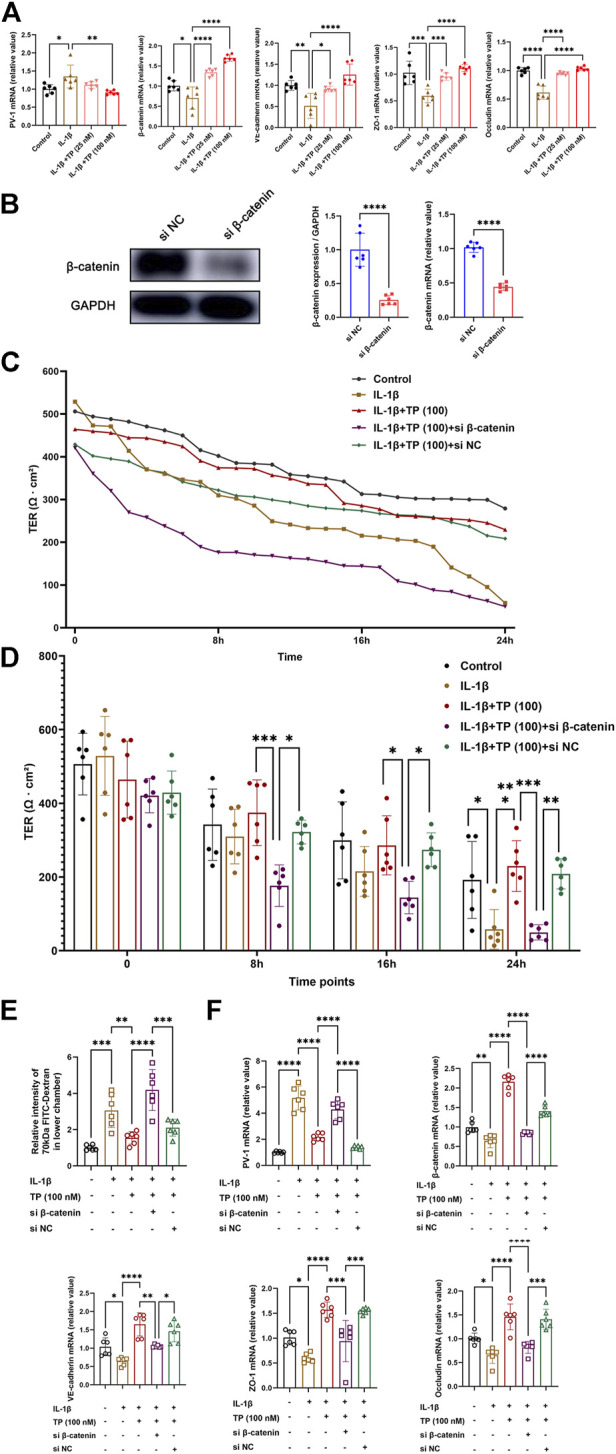
Terlipressin maintained the integrity of endothelial cell monolayer during IL-1β stimulation *via* β-catenin. Adherent human umbilical vein endothelial cells (HUVECs) were cultured in six-well plates and incubated with or without 10 ng/ml recombinant human interleukin-1β for 24 h **(A)** PV-1, β-catenin, VE-cadherin, ZO-1 and occludin mRNA expression with IL-1β and different doses of terlipressin (25 nM or 100 nM) treatment in HUVECs. After transfection with small interfering RNA for 24 h (si β-catenin or si Negative control), **(B)** cell lysates were immunoblotted with β-catenin to verify the efficiency and quantitative analysis, and **(C,D)** The integrity of endothelial cell monolayer was quantified by transendothelial electrical resistance assay (TER) at continuous time and indicated time points. **(E)** At 24 h after treatment in each group, the 70 kDa FITC-dextran permeability of HUVECs barrier integrity was measured. **(F)** PV-1, β-catenin, VE-cadherin, ZO-1 and occludin mRNA expression after transfection with small interfering RNA. *n* = 6. Data were expressed by mean ± SD. *p < 0.05, **p < 0.01, ***p < 0.001.

To further mimic the sepsis model *in vivo*, we stimulated HUEVCs with recombinant human IL-1β *in vitro*. Recombinant human IL-1β upregulated mRNA levels of PV-1, while downregulated β-catenin, VE-cadherin, occludin and zonula occludens 1 (ZO-1). Terlipressin (100 nM) significantly restored the tight and adhesin junction proteins (Figure 6A). siRNA targeting β-catenin successfully made β-catenin knockdown (Figure 6B). Compared with control, interleukin-1β stimulation resulted in increased endothelial permeability, and terlipressin treatment restored TER of endothelial cells, which β-catenin knockdown sharply demolished at the indicated time points (Figures 6C,D). The permeability was determined by measuring the passage of 70 kDa FITC-Dextran across HUVEC monolayers. The analysis showed that terlipressin decreased the endothelial permeability in HUVEC. β-catenin knockdown induced a 2-fold increase in the permeability of the monolayer compared to the IL-1β+TP group (Figure 6E). Moreover, β-catenin knockdown significantly decreased the mRNA levels of junction and adhesin proteins and increased PV-1 (Figure 6F).

## Discussion

**FIGURE 7:**
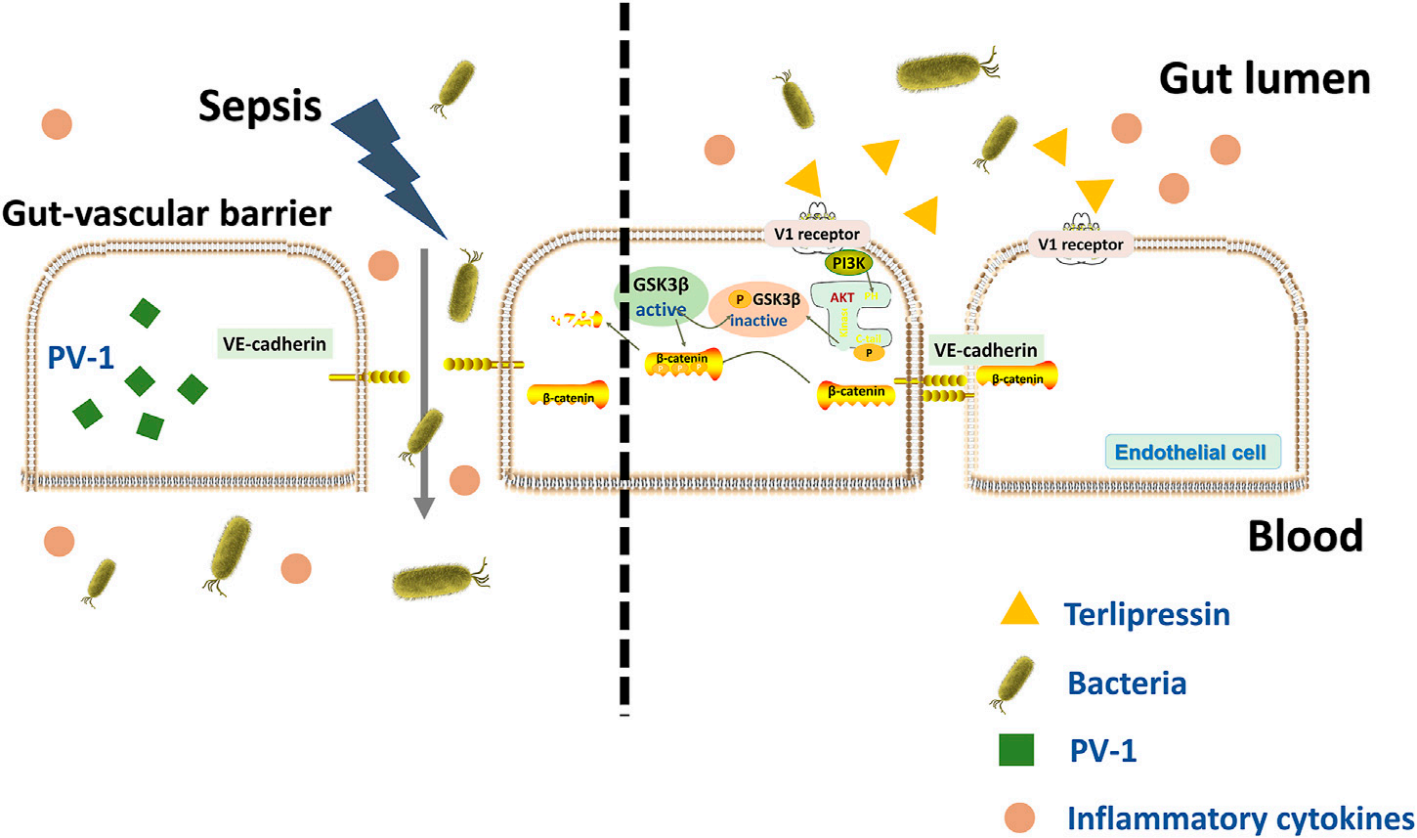
A schematic outlining of terlipressin protecting GVB from bacterial translocation into blood and TP/V1/PI3K/β-catenin signaling pathway in sepsis.

The current study demonstrated that severe disruption of GVB occurred after sepsis insult, and a protected GVB conferred by terlipressin could independently block bacterial translocation *via* blood vessels and subsequently reduce distant organ injuries, although bacteria crossed other intestinal barriers. The PI3K/β-catenin signaling might be involved in the protective effects induced by terlipressin (Figure 7).

The intestinal tract is regarded as the “motor” of critical diseases (Mittal and Coopersmith 2014). We have confirmed that the migrating protein and bacteria through bloodstream resulted in distant hepatic and renal injuries after intestinal I/R (Wen, Li et al., 2020; Lai, Zhan et al., 2021). GVB serves as the last firewall to limit the dissemination of intestinal toxins *via* the blood circulation (Spadoni, Zagato et al., 2015; Spadoni, Pietrelli et al., 2016). Increased intestinal vascular permeability in septic model was reported in previous literatures (He, Yuan et al., 2018; Li et al., 2020b). In the present study, we furtherly explored the change of GVB damage according to the data of two specific biomarkers (PV-1 and VE-cadherin) at different time point after CLP. The results showed that the destruction of GVB reached its peak at 6 h after CLP and then gradually recovered (Figure 1), indicating that a timely intestinal vascular protection is of important for relieving distant organ damages in gut-derived sepsis. Therefore, in this study, terlipressin 0.15 mg kg^−1^ was administered at 5 min after CLP challenge. Based on a classical conversion of drug dose from human to animal, this dosage of terlipressin in mice is approximately equal to 0.017 mg kg^−1^ in human, which has been commonly applied in the clinical settings (Treschan and Peters 2006; Reagan-Shaw, Nihal et al., 2008). The use of terlipressin significantly improved GVB damage (Figure 2) and reduced the translocation of macromolecules and bacteria (Figure 3) in mice after CLP, and raised TER in adherent endothelial cells (Figure 6). Consistent with our finding, He et al. demonstrated that early treatment with a selective V1a receptor agonist inhibited vascular leakage in ovine with septic shock (He, Su et al., 2016). Based on our findings in this study, terlipressin may be a preferable vasoconstrictor in gut-derived sepsis in the clinical settings. Moreover, based on the definite protective effect of terlipressin on GVB, the therapeutic possibilities of terlipressin for GVB damage induced by bacterial or viral intestinal infections, inflammatory bowel disease and other pathogenic challenges deserve to be explored seriously. Actually, several authors had investigated the therapeutic effects of terlipressin in spontaneous bacterial peritonitis (Lee, Han et al., 2009), gastrointestinal bleeding (Seo, Park et al., 2014) and hepatorenal syndrome (Uriz, Ginès et al., 2000).

It has been demonstrated that gut microbiome, mucus and mucosal barrier limit intestinal bacterial translocation and subsequently alleviate liver injuries (Albillos, de Gottardi et al., 2020). In this study, to furtherly clarify the limiting effect of GVB in various gut barriers, we examined the fluorescence of macromolecules in intestinal tissues and distant organs and analyzed the correlations between intestinal PV-1 expression and fluorescence intensity. The results showed that the level of GVB damage was closely associated with the expression of macromolecules in different organs, and surprisingly, numerous macromolecules were detected in serum and distant organs but not in ileum and colon due to the impaired GVB caused by CLP, whereas the protected GVB conferred by terlipressin efficiently prevented macromolecules in the gut lumen from transferring to distant organs *via* blood circulation even though the macromolecules had crossed other barriers and entered the intestinal tissues (Figure 3). Moreover, in line with previous findings (Spadoni, Zagato et al., 2015), the protected GVB prohibited entry of the microbiota into liver and lung (Figure 3), and then improved multiple organ injuries and survival after CLP (Figure 4). To the best of our knowledge, our notable findings for the first time demonstrate that GVB plays a critical role in blocking the development of gut-derived sepsis, and suggest that a healthy GVB can independently inhibit intestinal bacterial translocation *via* circulation and terlipressin may be a promising vasoactive agent for the critical patients with intestinal impairments because of its protective property of GVB.

It has been demonstrated that activation of the PI3K-Akt-Rac1 signaling can maintain the integrity of the blood-brain barrier (Wu, Chen et al., 2017) and our previous study showed that intravenous infusion of terlipressin markedly increased the expression of PI3K and p-Akt in ileal mucosa in rat’s intestinal ischemia model (Liu et al., 2020b). Hence LY294002, a specific inhibitor of PI3K, was used following terlrpressin treatment and p-Akt expression in ileum was examined in this study. Our results clearly showed that terlrpressin protected against gut-derived sepsis mainly through PI3K/Akt signaling (Figures 2–5). The PIK3/Akt/GSK3 is a classic signaling pathway and involved in many biochemical processes (Duda, Akula et al., 2020). GSK-3β is a key regulator for β-catenin expression and phosphorylation of GSK-3β can inhibit the degradation of β-catenin (p-β-catenin) and then increase β-catenin expression (MacDonald, Tamai et al., 2009; Nong, Kang et al., 2021). The current application of terlipressin increased p-GSK-3β and β-catenin expression, but decreased p-β-catenin in intestinal tissues (Figure 5). β-catenin is essential for reducing the vascular permeability and bacterial penetration (Spadoni, Zagato et al., 2015; Mouries, Brescia et al., 2019; Zhang, Chang et al., 2022). Several studies demonstrated that upregualtion of β-catenin could improve GVB in pathologic conditions (Birdsey, Shah et al., 2015; Mouries, Brescia et al., 2019; Zhang, Chang et al., 2022). When we knocked down β-catenin in endothelial monolayer, the increased TER, tight and adhesin junction proteins and decreased permeability across HUVEC monolayers conferred by terlipressin sharply changed (Figure 6). Whereas p-β-catenin caused adherence junction disruptions and cytoskeleton rearrangement, and then increased vascular endothelial hyperpermeability (Weng, Yu et al., 2019). Therefore, we suggested that activation of the PI3K/β-catenin pathway might be involved in the protective effect of terlipressin on GVB.

There were some possible limitations in this study. First, because knock-out/knock down models are missing, we have not figured out the mechanism of GVB damage induced by gut-derived sepsis. Further studies are needed to explore the causative mechanism and to develop the corresponding treatment. Second, the impact of β-catenin on GVB maintenance is still controversial (Grander, Grabherr et al., 2020). The definite signaling pathway of terlipressin for GVB protection is needed to be investigated. Third, the effects of terlipressin on GVB and bacterial translocation in sepsis need to be confirmed in translational researches and clinical trials. Final, hemodynamic improvement caused by terlipressin might be partly responsible for the reduction of organ injuries and animal mortality in our experiments, hence another vasoconstrictor, such as norepinephrine, should be set as the positive control.

Taken together, the present study reveals that sepsis leads to severe GVB disruption, and a vasoconstrictor, terlipressin, reduces GVB damage *via* PI3K/β-catenin signaling. The protected GVB conferred by terlipressin effectively prevents macromolecules and bacteria in the gut lumen from transferring to distant liver and lung *via* blood circulation and subsequently improves organ injuries and mortality after sepsis insult. Based on the key role of GVB in gut-derived sepsis, terlipressin may be a promising direction for the critical ill patients with gut-derived sepsis.

## Data Availability

The original contributions presented in the study are included in the article/Supplementary Material, further inquiries can be directed to the corresponding authors.
