# Supplementary material for: Improvement of gut-vascular barrier by terlipressin reduces bacterial translocation and remote organ injuries in gut-derived sepsis
Source: Front Pharmacol. 2022 Oct 7;13:1019109. doi: 10.3389/fphar.2022.1019109 (PMC9585222; doi:10.3389/fphar.2022.1019109)
Supplement: Supplementary file 1 [file DataSheet1.docx]

Supplementary Material

# Supplementary Table

**Supplementary Table 1. Oligonucleotide Primer Sequences Used for Quantitative Polymerase Chain Reaction**

| **Gene** | **Forward primer sequence****s (5’-3’)** | **Reverse primer sequences (5’-3’)** |
| --- | --- | --- |
| *IL-6 (mice)* | CTGCAAGAGACTTCCATCCAG | AGTGGTATAGACAGGTCTGTTGG |
| *IL-1β (mice)* | GAAATGCCACCTTTTGACAGTG | TGGATGCTCTCATCAGGACAG |
| *TNF-α (mice)* | CAGGCGGTGCCTATGTCTC | CGATCACCCCGAAGTTCAGTAG |
| *PV-1 (mice)* | GCTGGTACTACCTGCGCTATT | CCTGTGAGGCAGATAGTCCA |
| *β-actin (mice)* | GTGACGTTGACATCCGTAAAGA | GCCGGACTCATCGTACTCC |
| *PV-1 (human)* | GCTGCTGGTATTACCTGCG | GCCATAGACCATGAAGAGCAC |
| *β-catenin(human)* | AAAGCGGCTGTTAGTCACTGG | CGAGTCATTGCATACTGTCCAT |
| *VE-cadherin (human)* | TTGGAACCAGATGCACATTGAT | TCTTGCGACTCACGCTTGAC |
| *Occludin (human)* | ACAAGCGGTTTTATCCAGAGTC | GTCATCCACAGGCGAAGTTAAT |
| *ZO-1 (human)* | CAACATACAGTGACGCTTCACA | CACTATTGACGTTTCCCCACTC |
| *GAPDH (human)* | GGAGCGAGATCCCTCCAAAAT | GGCTGTTGTCATACTTCTCATGG |

# Supplementary Figures

## Supplementary Figure 1


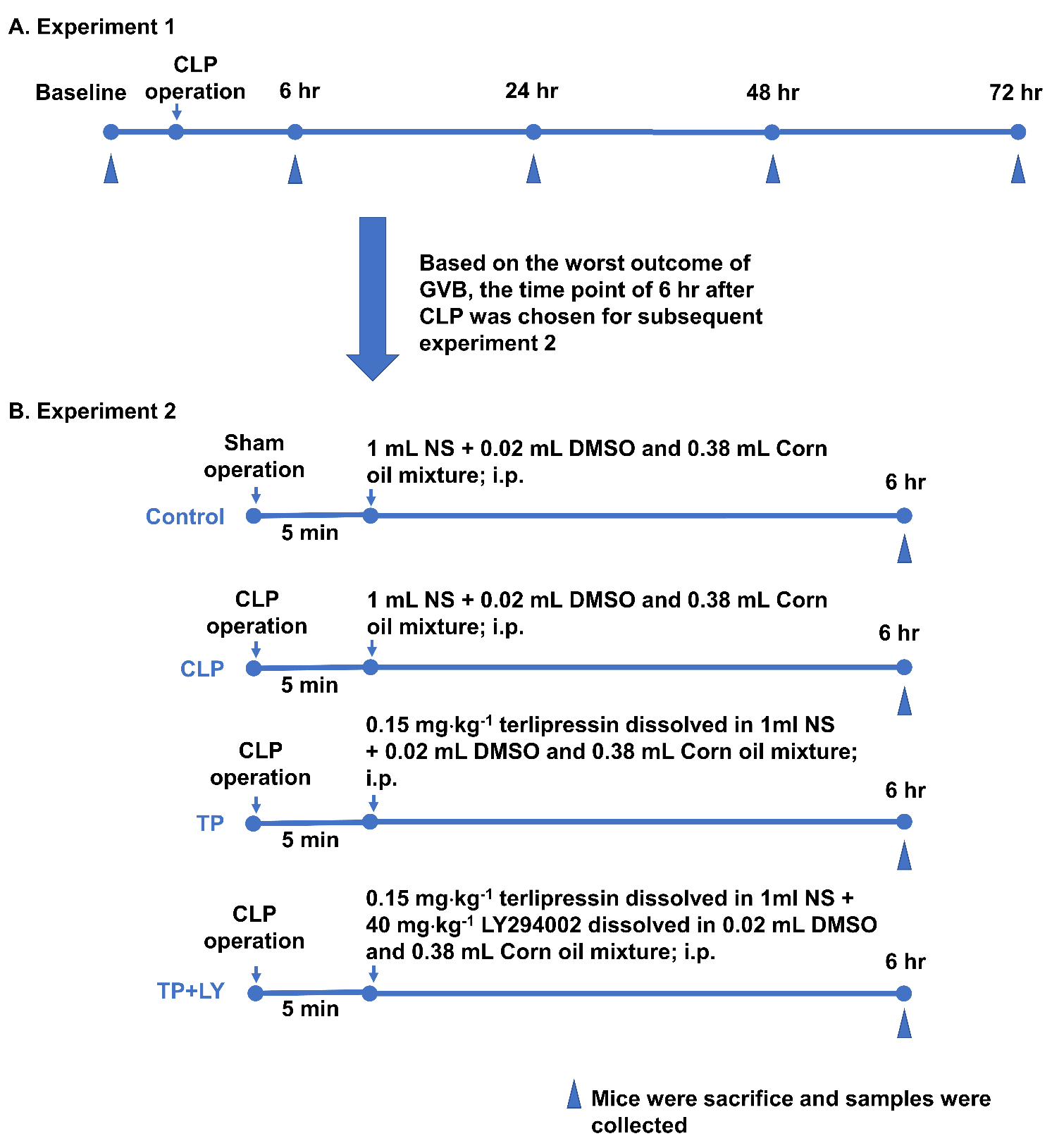


**Supplementary Figure 1.** Detailed illustration of animal groups and treatments.

## Supplementary Figure 2


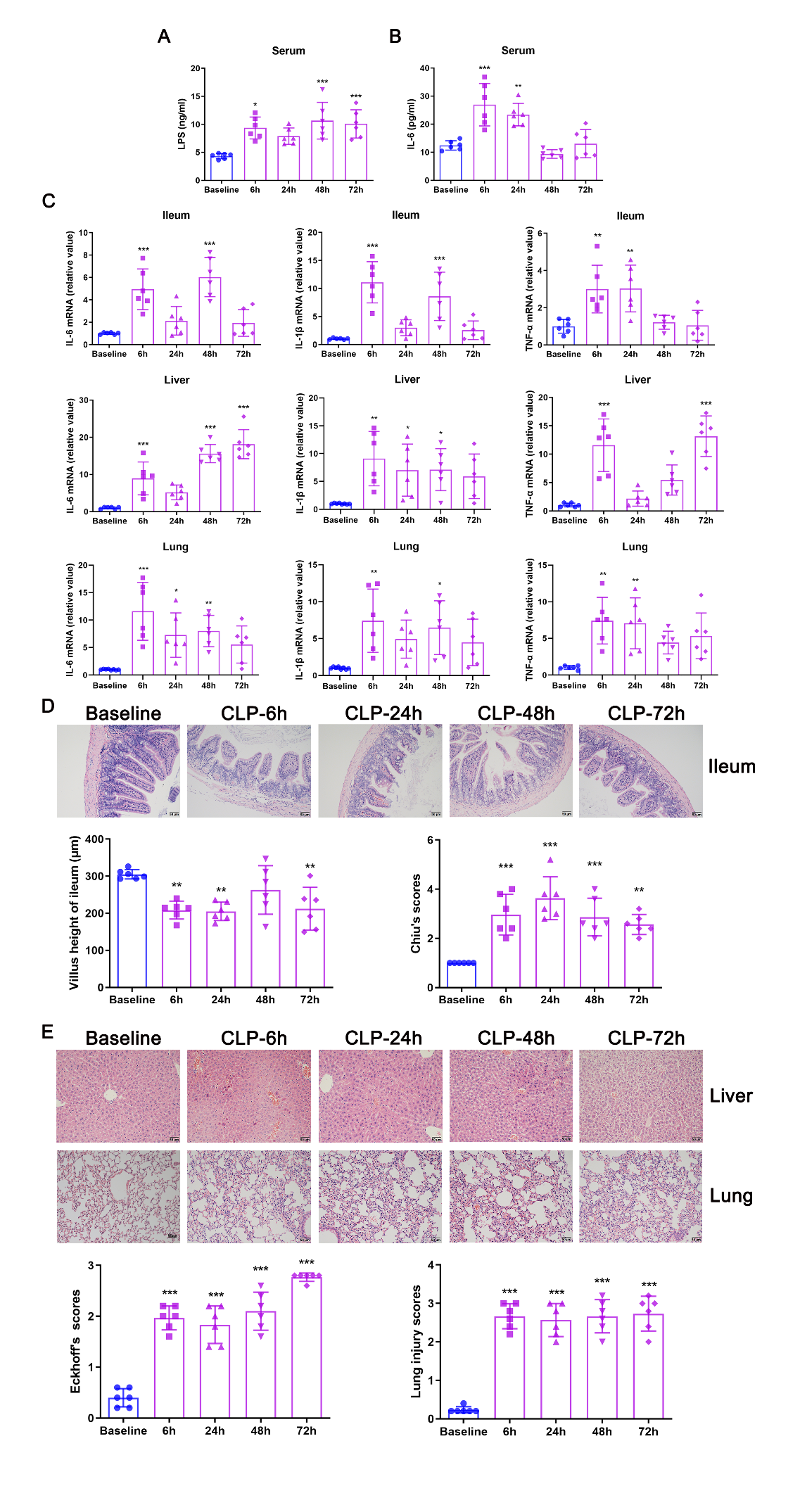


**Supplementary Figure 2.** **Cecal ligation and puncture (CLP) induced inflammation and organ injuries.** C57BL/6J male mice were respectively euthanized after sham operation or at 6 hr, 24 hr, 48 hr and 72 hr after CLP operation. (A, B) Serum interleukin (IL)-6 and lipopolysaccharides (LPS) levels were determined by enzyme-linked immunosorbent assay (ELISA), n=6. (C) IL-6, IL-1β or tumor necrosis factor (TNF)-α mRNA levels in ileum, liver and lung, n=6. (D) The ileum (×200) histological images (hematoxylin-eosin, HE staining), Chiu’s scores and villus height of ileum, n=6. (E) The histological images (×200) of liver and lung and morphological injury scores, n=6. Scale bars: 50 μm. Data were expressed by mean ± standard deviation. **P* < 0.05, ***P* < 0.01, *** *P* < 0.001 vs Baseline group.
